# Sources, Chemical Functionalization, and Commercial Applications of Nanocellulose and Nanocellulose-Based Composites: A Review

**DOI:** 10.3390/polym14214468

**Published:** 2022-10-22

**Authors:** Danish Tahir, Muhammad Ramzan Abdul Karim, Hong Hu, Sufyan Naseem, Muhammad Rehan, Mairaj Ahmad, Minglonghai Zhang

**Affiliations:** 1 School of Fashion and Textiles, The Hong Kong Polytechnic University, Hung Hom, Hong Kong, China; 2 Faculty of Materials and Chemical Engineering, Ghulam Ishaq Khan Institute of Engineering Sciences and Technology, Topi 23640, Pakistan; 3 Department of Mechanical Engineering, The Hong Kong Polytechnic University, Hung Hom, Hong Kong, China; 4 Department of Industrial and Systems Engineering, The Hong Kong Polytechnic University, Hung Hom, Hong Kong, China; 5 Department of Chemical Engineering, Materials, Environment (ICMA), University of Rome “La Sapienza”, Via Eudossiana, 18, 00184 Rome, Italy

**Keywords:** nanocellulose, biodegradable, nanocomposites, chemical functionalization, extraction, applications

## Abstract

Nanocellulose is the most abundant material extracted from plants, animals, and bacteria. Nanocellulose is a cellulosic material with nano-scale dimensions and exists in the form of cellulose nanocrystals (CNC), bacterial nanocellulose (BNC), and nano-fibrillated cellulose (NFC). Owing to its high surface area, non-toxic nature, good mechanical properties, low thermal expansion, and high biodegradability, it is obtaining high attraction in the fields of electronics, paper making, packaging, and filtration, as well as the biomedical industry. To obtain the full potential of nanocellulose, it is chemically modified to alter the surface, resulting in improved properties. This review covers the nanocellulose background, their extraction methods, and possible chemical treatments that can enhance the properties of nanocellulose and its composites, as well as their applications in various fields.

## 1. Introduction

Polymeric cellulosic materials with high biodegradability and eco-friendliness have received a lot of attention, owing to the damage caused by petroleum base products, such as global warming, green gas emissions, and many others [1]. Several researchers are working on cellulosic fibers, from which nanocellulose can be extracted. The use of nanocellulose as a reinforcement in composites is because of their mesoscopic properties [2]. Nanocellulose is derived from plant cell walls and has extremely valuable properties, such as high surface area and strength [3,4,5]. Furthermore, the nanocellulose surface is easy to modify because of the large number of hydroxyl groups present in its structure. Nanocellulose has numerous applications in our daily lives, including filtration membranes, food packaging, biomedical, and so on [6].

Various review papers on nanocellulose extraction and their application have already been published [7,8,9], but in the present review, we described nanocellulose extraction by comparing the various chemical and mechanical extraction methods, chemical functionalization of cellulose, and cellulose composites processing, as well as their application in various fields. Chemical modification of cellulose is important for achieving the strong interfacial bond between cellulose and the matrix; therefore, the detailed discussion on chemical modification and the different techniques used for functionalizing the cellulose surface is also part of this review. Cellulose nanocomposite processing is also of prime importance for scientists working in the field of biocomposites, and numerous techniques can be used to manufacture cellulose composites. The second part of the review analyzes the nanocellulose composite production methods, followed by the applications of composites in various fields, such as biomedical, paper, coatings, and water treatment.

## 2. Nanocellulose and Its Various Sources

**Figure 1:**
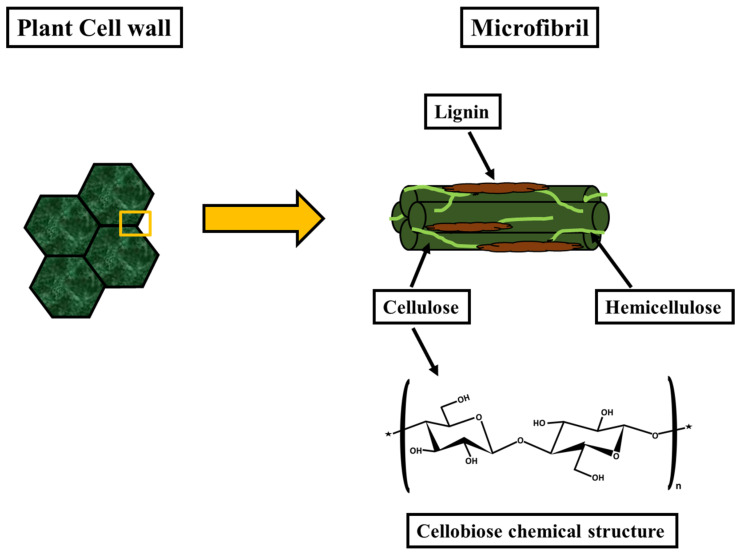
Plant cell wall structure and cellobiose chemical structure.

As shown in Figure 1, the cell walls of most plants contain hemicellulose, cellulose, and lignin. Lignin acts as a binder between cellulose and hemicellulose, holding them together. It has high stiffness and strength and can protect the cell wall from the outside environment. The amount of lignin in the plant cell wall ranges from 10 to 25% by weight, while the amounts of hemicellulose and cellulose are 20–35% and 35–50%, respectively [1,10,11]. The main component of the cell wall is cellulose, composed of repeating units of cellobiose, linked together with β-1,4 linkages, as shown in Figure 1. Intermolecular or intermolecular hydrogen bonding is used to connect the repeated units. Bonding occurs between the same or different chains via open hydroxyl groups [12]. Hemicelluloses are primarily xylans and glucomannans, connected by short or branched chains. Hydrogen bonding plays a vital role in providing compactness, strength, and solvent impermeability to the networks in cellulose fibers.

Strong hydrogen bonding networks and a variety of hydroxyl groups give cellulose fiber exceptional physical and mechanical qualities. The orderly packing of the chain molecules in the crystalline parts promotes high stiffness, whereas the amorphous parts give flexibility to the bulk material [13]. For general lignocellulosic biomass, the cellulose fibers present in between the crystalline and amorphous regions have a diameter of 3–100 μm and a length of 1–4 mm [14].

Nanocellulose fibers with a diameter of less than 100 nm and a length in the micrometers range deserve special attention. Nanocellulose fibers are transparent and rich in hydroxyl groups. These groups have a reactive surface that can be modified to obtain the desired properties [15]. Nanocellulose nanofibers have a low density of 1.6 g/cm^3^ and has exceptional strength [14]. Additionally, they have a tensile strength of nearly 10 GPa and a high strength-to-weight ratio that is eight times greater than stainless steel. 

The three primary forms of nanocellulose materials are BNC, NFC, and CNC, as shown in Table 1. Three cellulosic forms have unique qualities, including biodegradability, tunable surface chemistry, barrier properties, non-toxicity, high mechanical strength, crystallinity, and high aspect ratio. Such a remarkable nature of nanocellulose makes it a new material for food packaging and fillers in composites [16,17]. High-strength nanocellulose, sometimes referred to as CNC, is typically recovered by the process of acid hydrolysis from cellulose fibrils [18]. It is shaped like a short rod or a whisker and has a diameter of 2–20 nm and a length of 100–500 nm. Additionally, it is entirely composed of cellulose, with high crystallinity ranging from 54 to 88%. The long and entangled nanocellulose that may be mechanically removed from cellulose fibrils is another type of nanocellulose and is known as NFC, often referred to as micro-fibrillated cellulose. Its size ranges from 500 to 2000 nm in length and 1–100 nm in diameter [19,20]. It is made from 100% cellulose, with crystalline and amorphous region parts. Another different type of nanocellulose is BNC. It is formed by bacteria, primarily *Gluconacetobacter xylinus*, over a few days to two weeks, whereas lignocellulosic biomass is the primary constituent for the extraction of CNC and NFC (top-down method). As BNC is extracted from bacteria, other amorphous compounds, such as lignin, hemicellulose, and pectin, are never present in the pure form of BNC [21,22]. The chemical makeup of BNC is identical to that of the other two types of nanocellulose.

## 3. Nanocellulose Extraction Processes

**Figure 2:**
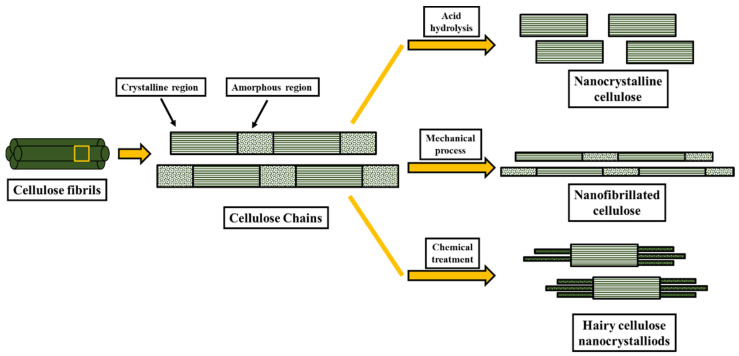
Schematic showing nanocellulose extraction from lignocellulosic biomass.

The use of agricultural leftovers for the extraction of nanocellulose is a very appealing field from the researcher’s point of view. Agriculture leftovers are lignocellulosic biomass that are rich in cellulosic content. The extraction of nanocellulose from lignocellulosic biomass involves various steps that can be seen in Figure 2. First, the pretreatment eliminates the non-cellulosic compounds, including lignin, hemicellulose, pectin, etc., followed by nanocellulose extraction through various extraction techniques [23].

### 3.1. Biomass Treatment for Nanocellulose Extraction

The pretreatment of lignocellulosic biomass to remove the amorphous compounds is the first step in the extraction of nanocellulose. Alkali treatment and acid-chlorite treatment are the two common methods for pretreating biomass. The amorphous compounds are removed from the biomass in the process of alkali treatment, primarily by treating the biomass with an alkali solution. Alkali can be sodium hydroxide or potassium hydroxide. Several scholars have provided in-depth descriptions of the alkaline pretreatment process [24]. Sharma et al. [25] worked on an alkali treatment technique to extract cellulose nanofibers from rice straw waste. For the removal of hemicelluloses and lignin, the rice straw was heated to 90–160 °C for 1–2 h after being soaked in various NaOH concentrations (8–16%).

Acid-chlorite treatment is another technique used for the removal of amorphous compounds. The simultaneous treatment of sodium chlorite, along with glacial acetic acid as an acidifying agent, removes the majority of the lignin from the lignocellulosic biomass. This procedure is also referred to as a bleaching or delignification technique. It is carried out when a mixture of lignocellulosic biomass and distilled water is added to the sodium chlorite and acetic acid solution at 70–80 °C for 4–12 h [26]. After completion of the treatment, the mixture is stirred continuously for an entire night before being washed with distilled water to bring the pH level to a neutral state. This leads to a collection of white residue, which is then dried at 50 °C in an oven to obtain lignin-free holocellulose [27].

### 3.2. Nanocellulose Isolation

Acid hydrolysis, mechanical treatment processes, and enzymatic hydrolysis are some of the techniques used for nanocellulose isolation. Table 2 shows three cellulose isolation processes. The most commonly used technique for extracting nanocellulose is acid hydrolysis [28]. It involves the use of strong acids, such as sulfuric acid, that can easily hydrolyze the amorphous area of cellulose fibrils by esterifying the hydroxyl groups with sulfate ions [29,30]. Maiti et al. [31] suggested the use of 47% of sulfuric acid to recover nanocellulose from waste tissue papers, China cotton, and south African cotton. The results show that the types of the precursors and the hydrolysis conditions largely determine the shape and size of the nanocellulose. It was noted that esterification creates a colloidal dispersion of crystalline nanocellulose inside the reaction mixture. Acid hydrolysis can be performed by other mild acids, such as formic acid, phosphoric acid, etc. Bond cleavage is the mechanism behind the acid hydrolysis of cellulose. The glycosidic bonds present between anhydroglucose units are subjected to hydrolytic cleavage, resulting in the rearrangement of tangling chains and strain release. Acid hydrolysis thereby dissolves the amorphous portion, leaving the crystalline sections intact. Formed crystalline regions are then projected to mechanical treatment, which transforms them into fine cellulose particles. The use of a 30–50% concentration of sulphuric acid in acid hydrolysis gives very fine cellulose particles. Reinforcing fine cellulose particles in biocomposites facilitates high plastic deformation in the composite. Acid hydrolysis reactions and the properties of nanocellulose can be controlled by varying the concentration, time, and temperature provided [32]. The size of cellulose particles and their distribution depend highly on the temperature, time, and concentration of the acid. A high temperature and short reaction time are usually recommended for dilute acid hydrolysis. Using dilute acid eliminates the need for acid recovery. Iranmahboob et al. [33] performed dilute acid hydrolysis and found that, to achieve a high yield from dilute acid, high temperature, less time, and high pressure are required. Similarly, Hamelinck et al. [34] performed hydrolysis using concentrated acid and concluded that hydrolysis through concentrated acids requires moderate temperatures and longer reaction times. The use of concentrated acid enhances the difficulty of recovering acid from the mixture. Later stages of acid hydrolysis also involve the washing of cellulose, which is usually achieved by centrifuging the mixture of cellulose and cold water [35]. The main disadvantage of this procedure is the need to treat acid-containing wastewater before releasing it into the environment.

Enzymatic hydrolysis comes under the list of biological processes that use enzymes to break down the cellulose fibers into washed cellulose [36]. The literature reveals that cellulase, cellobiohydrolase, endoglucanase, etc., are frequently used enzymes for this process. Although the mechanism is intricate, the enzyme’s activity is dependent on catalyzing the breakage of the connecting H-bond in between the cellulosic fibers [37]. Enzymes activity involves the removal of hemicellulose, protection of cellulose from hydrolysis, and production of monosaccharides from hemicellulose for subsequent fermentation to produce bioethanol [38]. The cellulases and hemicellulases present in the process are closely connected to effectively hydrolyze a variety of lignocellulosic biomasses. When compared to acid hydrolysis, it is normally conducted under milder conditions and takes significantly longer to operate. To reduce the processing time, enzymatic hydrolysis can be used in combination with other techniques. Moniruzzaman et al. [39] used a novel method to separate nanocellulose from wood by pre-treating the cellulose with an ionic solution, followed by laccase-enhanced enzymatic hydrolysis. A comparison of the produced nanocellulose to the existing methods of cellulose preparation shows that the produced cellulose has a high surface area and higher crystallinity with enhanced thermal properties.

**Figure 3:**
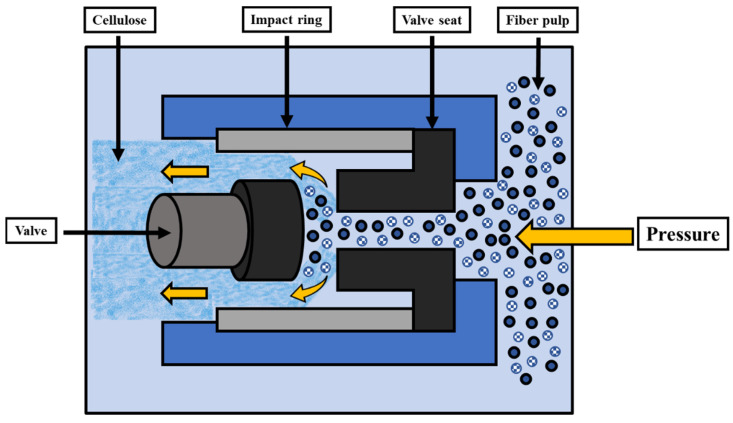
Schematic for HPH.

Different mechanical techniques, including ball milling, ultrasonication, and high-pressure homogenization (HPH), can be used to mechanically prepare nanocellulosic fibers [17]. However, these methods require a lot of energy, which is why some pre-treatment is always suggested to save energy. HPH involves the treatment of a cellulose mixture at high pressure and velocity [36]. High pressure and velocity divide the cellulose microfibrils into nanometre-sized fragments, based on the fluid shear and impact forces created. Figure 3 shows the schematic of the homogenizer used for cellulose isolation. Li et al. [40] used HPH to separate nanocellulose from sugarcane bagasse. To dissolve the bagasse cellulose, the material was initially pre-treated with an ionic liquid (1-butyl-3-methylimidazolium chloride ([Bmim]Cl)), followed by passing the homogenized solution at high pressure, without becoming clogged. The produced nanocellulose had a diameter of 10–20 nm, with a crystallinity lesser than the original cellulose. By using HPH, Wang et al. [41] separated nanocellulose from cotton cellulose. The final product obtained (20 nm in diameter) was less thermally stable and had a low crystallinity than the pre-treated cotton cellulose. A decrease in crystallinity is because of the high pressure, which disrupts cellulose intermolecular and intramolecular hydrogen bonding.

Cellulose fiber can also be defibrillated using the ultrasound’s hydrodynamic forces, through the process of ultrasonication [42]. In this technique, ultrasonic energy is generated by mechanically oscillating the power and implosion of gas bubbles. The generated energy is then absorbed by the liquid molecules [43,44]. Tang et al. [45] used the process of ultrasonication to extract the nanocellulose from the wood pulp. The results showed that cellulose obtained without ultrasonication has a yield of 48.16%, whereas ultrasonication of the sample gives the cellulose sample a yield of 85.38%. The obtained nanocellulose had widths of 10–100 nm and a yield of 85.38%. 

**Figure 4:**
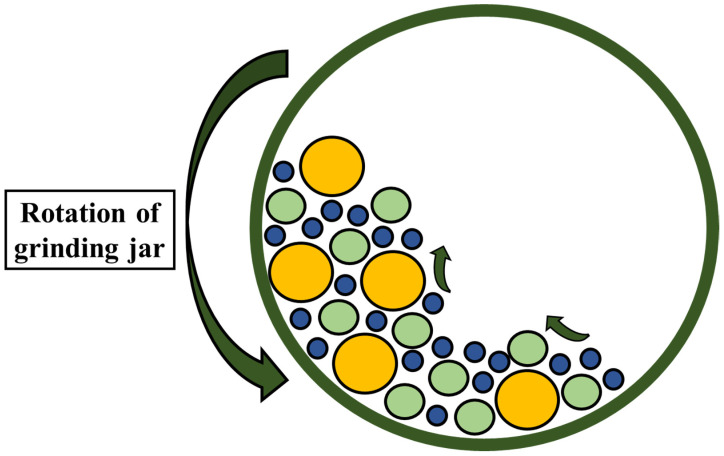
Schematic for planetary ball mill.

Cellulose fibers are defibrillated using another mechanical technique, which involves the use of ball milling. Since the 1990s, researchers have used ball milling for grinding and improving particle size. The milling jar contains milling balls of various sizes, as one of the primary components of the ball milling machine. Planetary ball mills and vibration ball mills are some of the common types of ball mills used in industry and laboratories today [46]. The planetary ball mill is the one that defibrillates cellulose and biomass the most frequently. In the planetary ball, the mill balls collide with each other and with the wall of the milling jar, thus creating friction, which helps in size reduction [47,48]. The created friction grinds the large-size materials into smaller particles with large surface areas. The friction is linked with the shear forces that are produced between the balls and the surface of the rotating jar as a result of the centrifugal force [49,50]. The application of shear force onto the cellulose breaks them into nano-size particles. The number and size of the balls, time, weight ratio between the balls and material, and speed are a few of the variable elements on which the characteristics of ball-milled products depend [51]. The planetary ball mill is also shown in Figure 4. Ago et al. [46] investigated the properties of ball-milled, cotton-derived cellulose at 400 rpm for two hours by varying the water content in the mixture. It was discovered that the presence of a small amount of water (10 wt.%) in a dry state changes the cellulose type I to amorphous. However, when water is up to 30 wt.% cellulose type I, it transformed into the stable form of cellulose type II. This suggests that the crystalline structure of the cellulose is greatly influenced by the amount of water in the milling jar [52].

### 3.3. BNC Extraction

Besides plants, bacteria can be used to produce cellulose. Bacterial cellulose can be used as a primary source for the production of CNC and cellulose nanowhiskers because of their high purity and crystallinity. It is generally acknowledged that the source of the bacterial cellulose and the isolation techniques utilized affect the shape of BNC. Some commonly used bacteria are *Pseudomonas, Rhizobium, Sarcina, genera Acetobacter, Azotobacter,* and *Alcaligenes.* *Acetobacter xylinum*, a species of bacteria that produces acetic acid, is the most effective generator of BNC. Cellulose biosynthesis is the method of extraction of BNC. Extracted BNC has a width of less than 100 nm and is 100–1000 nm long [53,54]. BNC isolation from bacterial cellulose can be achieved by using acid hydrolysis, enzymatic hydrolysis, and ionic liquids. By acid hydrolysis, CNC and BNC can likewise convert into bacterial nanocrystals. However, acid hydrolysis also has some disadvantages, as it decreases the degree of polymerization (DP) and reduces the number of sulphate-containing nanocrystals. Reduction in DP and nanocrystals brings down the mechanical properties of cellulose nanocomposites. Therefore, the enzymatic system is suggested to retain the actual properties of bacterial cellulose. Ullah et al. [55] developed a cell-free enzyme system for producing bio-cellulose. The system was developed using a single-cell line and contained all the enzymes needed to run a successful biosynthesis process. The prepared bio-cellulose were scattered and had extracellularly produced glucose chains. The results revealed that a better yield can be obtained by following the produced cell-free system. 

## 4. Chemical Treatment of Nanocellulose

**Figure 5:**
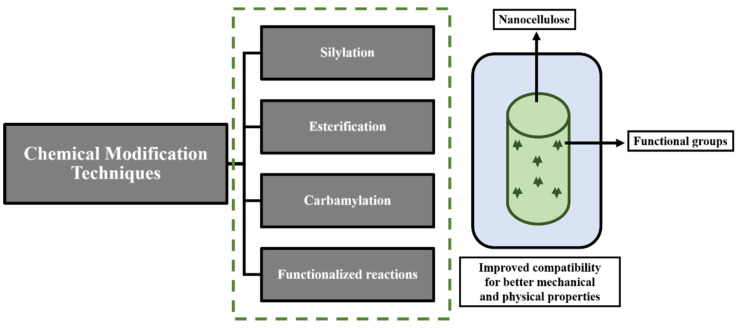
Scheme for various nanocellulose modification methods.

Surface modification of nanocellulose is one of the important steps in nanocomposite preparation that can improve the mechanical performance of cellulose nanocomposites. Pretreatment using some chemicals is, therefore, required to strengthen the interfacial bond between the matrix and cellulosic material. Treatment also provides a barrier against moisture absorption by making a good interfacial bond that restricts the water movement towards the interface and enhances the wetting properties of the reinforcement towards the matrix [56,57,58,59]. Figure 5 shows the scheme of various chemical modification processes. One of the popular techniques for the surface modification of cellulose is the attachment of covalently bonded hydroxyl groups on the surface of nanocellulose or direct chemical modification. Additionally, the modification of grafted polymers and nanocomposites is frequently achieved by the grafting of polymers onto biopolymer [60]. The creation of amphiphobic surfaces is one of the principal uses of surface-modified nanocellulose. Amphiphobic surfaces can be related to uses such as self-cleaning, anti-reflective, etc. Both polar and non-polar liquids can be protected by an amphiphobic surface [61,62]. Nanocellulose is also used to alter the surface’s wettability because it adds hydroxyl groups to the surface, enhancing the surface’s hydrophobization when exposed to chemicals. The hydroxyl groups in nanocellulose are also modified through other chemical processes, such as etherification, carbonylation, and silylation [63]. CNC is often chemically modified by poly(glutamic acid). To obtain the best properties, cellulose is partially oxidized, followed by the reaction of amino groups of poly(glutamic acid) with the aldehyde groups. Treatment with poly(glutamic acid) enhances the hydrophobicity of the cellulose, resulting in the formation of a strong interfacial bond with a matrix [64]. Table 3 describes the various chemical modification methods, their chemical sources, and the modified characteristics of cellulose obtained by the treatment.

The most effective chemicals for improving the interfacial bond between cellulose and matrix are silane coupling agents, such as alkoxy silane. Silane’s hydrocarbon chains increase the fibers’ wettability, raising their chemical affinity toward the matrix. Silane modification is highly efficient for alkali-treated fibers, rather than for untreated fibers. High efficiency is because of the creation of more reactive sites by silane treatment. Cellulose fibers can be projected to silylation when treated in the absence of water and at high temperatures. The unavailability of water at high temperatures restricts the Si–OR group to interact with the OH group of cellulose; therefore, the silane group comes in contact with the OH of the cellulose [70,71,72,73]. To start a response between the Si–OR and OH groups of cellulose, water content needs to be increased. Silylation effect can also be observed by using chlorodimethyl isopropylsilane on NFC that is extracted from blanched softwood mash. Surface analysis shows that silylation causes the surface of cellulose nanofibers to acquire substituted silyl groups, which are beneficial in the enhancement of hydrophobicity. 

Carbamylation is the method in which isocyanic acid binds to the functional groups of cellulose to modify its surface. Navarro et al. [67] used butyl 4-(Boc-aminomethyl) phenyl isothiocyanate to modify NFCs and used DMSO as the solvent. Additionally, NFCs were treated with a solution of rhodamine B ester, which was modified with N-hydroxysuccinimide to create luminous NFCs. Such kinds of NFCs are commonly used in sensor applications. 

The TEMPO oxidation technique, which has grown in popularity in the recently referenced publications, is another interesting way to chemically alter the surfaces of NFC. In this process, the NFC surface can initially be modified by attaching carboxyl groups to it. The attached groups then assist the other functionalization processes that are required to produce modified NFCs. Recent publications show that TEMPO-oxidized NFCs found numerous applications in water-resistant films, which can be employed as fluorescence sensors for the detection of nitroaromatics [68,69].

Acetylation and esterification are important approaches to modify the surface of NFCs [74]. It involves the utilization of both aromatic and aliphatic carboxylic reagents in organic media. Various types of research have been performed where acetylation is used as a surface modification technique [66,75,76,77,78]. Acetylation refers to the response of cellulose’s OH groups to acetyl groups, resulting in the plasticization of lignocellulosic strands [76]. Acidic anhydride is commonly used as an acetylation media and modification is achieved by preparing a solution of ethanol/toluene solvent and acidic anhydride, followed by the addition of NFC suspension. Bulota et al. [66] performed the acetylation modification process to study its effect on the properties of polylactic acid (PLA) and acetylated NFC composites. The acetylation procedure was conducted at 105 °C in toluene, which proved to be a successful method of boosting cellulose dispersion in a non-polar PLA solution. Acetyl content on the treated cellulose highly depends on the reaction time and could be assigned a degree of substitution (DS) of 0.43. Fourier transform infrared spectroscopy was also used to verify the acetylation. The findings show a prominent impact of nanofibers, with higher DS, on the characteristics of polylactic acid-acetylated NFC composites. Various anhydrides can be used to enhance the cellulose performance in cellulose-reinforced composites [79]. Recently, propionic anhydride has been used, instead of acetic anhydride, because it provides superior dimensional stability to cellulose. It is also noted that DS can be increased by combining pyridine with the catalyst sulphuric acid, whereas the grafting of acetyl moieties can increase the hydrophobicity of the cellulose. The combined effects of the aforementioned treatments led to the creation of NFCs with a highly hydrophobic surface that strongly restricts water movement toward the interface. Missoum et al. [80] used several anhydrides in an ionic liquid as a novel method for heterogeneous surface modification that lead to effectively nanoscaled cellulose substrate grafting, without modifying their morphological characteristics. Liquid–liquid extraction was employed to recycle the ionic liquid in consideration of environmental concerns. Utilizing these ionic liquids has the benefit of not producing volatile organic molecules.

Methylcellulose (MC) is a synthetically altered form of cellulose used as a reinforcement in nanocomposites. It is an ester of cellulose and has up to 32% of methoxy groups. MC with a methoxy content of 29.1% shows a DS of 1.75 [81]. Reinforcing nanocellulose with MC, or the use of MC as filler in biopolymer, can increase the ductility of the product. Hynninen et al. [82] prepared MC/CNC nanocomposite fibers by wet-spinning of nanocomposite hydrogels in an ethanol coagulation bath. The prepared fibers showed a high modulus, as well as ductility. Fibers with 80% of MC and 20% of CNC provided the best mechanical properties. An increase of CNC in fibers makes them brittle, whereas, to achieve the desirable properties, MC and CNC must be used hand-in-hand. The combination of MC with CNC provides a synergistic effect on the fibers, and no fiber can be prepared solely from CNC or MC.

## 5. Nanocellulose Composites and Their Processing

Natural fiber composites can be produced by various processing methods, such as resin transfer mold (RTM) [83], injection molding [84], and extrusion [85]. Three common factors, namely molding time, temperature, and pressure, can be used to optimize the composite in these manufacturing processes [86]. Some pre-fabrication steps are also required to achieve better properties. For example, preheating the natural fibers before processing them into composites is frequently used to lower their moisture content, but the strength of the composites could be significantly affected by the degradation of cellulose at high temperatures. Other than temperature, the inadequate distribution of cellulose fibers in the matrix may cause fiber aggregation, which results in a decrease in the composite’s tensile strength [87]. There are several methods for processing composite materials, but the majority of them are modifications of the fundamental procedures. The most crucial phase in the manufacturing of a composite is choosing the best technique. This decision can be made based on several factors, such as how simple it is to produce, the geometry of the product required, the maximum cost, the application, and the properties required [88]. Nanocellulose is usually obtained in aqueous form; therefore, the composite processing is also performed in some suitable aqueous medium. As a result, the manufacturing of composites was best served by using polymers that are water-soluble or have the ability to form dispersions. Several wet lab techniques for processing cellulose nanofiber-reinforced nanocomposites are in practice, but the most commonly used techniques are layer-by-layer assembly (LbL), freeze drying, electrospinning, cast-drying, etc. [89,90].

The most typical method for preparing thin composites and coatings is LbL assembly. Operation characteristics, including adhesion, gas barrier, and composition, should be tuned for adaptability throughout the processing [91]. For single molecular level nanoparticle deposition, various substrates can be used, and the deposition occurs through the involvement of hydrogen bonding, electrostatic interactions, and hydrophobic–hydrophobic interactions [92]. For nanocomposites, LbL technology can provide the use of numerous substrates, along with the precise control of biocomponent thickness and distribution [93]. Fibers, flat wafers, textiles, particles, glass, and flexible films can be assembled into nanocomposites using different deposition techniques, such as spin-coating, immersive/dip-coating, and spray-coating [94]. By choosing the suitable substrate and its dissolving solvent, the prepared composite can be separated from the substrate and used for additional post-processing purposes [95].

**Figure 6:**
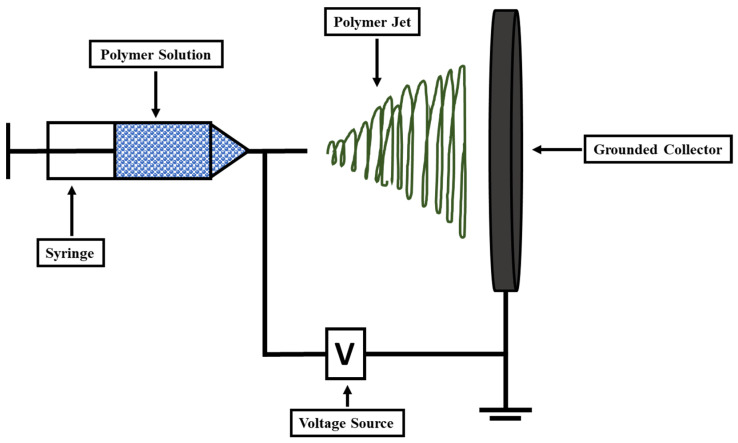
Schematic showing the process of electrospinning used for nanocellulose fiber preparation.

The fiber spinning processes, such as wet spinning and electrospinning, are some of the most common methods for creating nanocomposite fibers or biopolymer fibers [96]. Biopolymer microfibers are produced on a large scale via wet spinning, while electrospinning is utilized to produce mats from extremely fine nanofibers [97]. The fiber spinning techniques depends on the shear force acting along the fiber. These forces also help in aligning the nanostructured components during spinning [98]. Recently, the electrospinning technique has been employed by researchers to coat cellulose matrix with CNC. The orientation of the coated product is an important factor and can be monitored through uniaxial alignment [99]. The mechanical and thermal stability of the cellulose nanofibers can be improved by controlling the fiber’s orientation. The electrospinning technique is also shown in Figure 6.

**Figure 7:**
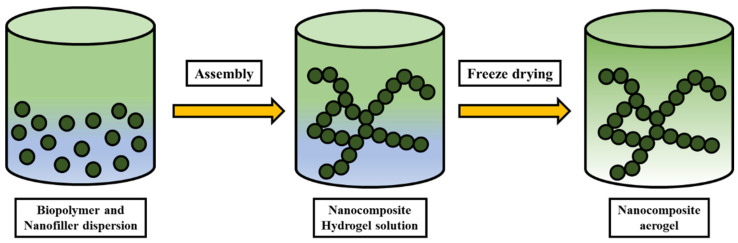
Freeze drying process for nanocomposite preparation.

The freeze-drying process is another method used to prepare nanocomposite aerogels from materials such as starch, cellulose, silk, alginate, and chitosan [100]. The schematic for the freeze-drying process is shown in Figure 7. This process is sometimes referred to as lyophilization because of the removal of water from suspensions of composite materials or aerogels. A sublimation process is usually used to freeze the material and involves the removal of small molecules from the solvent [101]. The aerogel particles’, pore size, and characteristics of the dispersion can be altered according to the requirement [102]. Altering the shape of the container can also affect the aerogel’s formation and orientation. The method of preparing aerogel using an ice template is most commonly used in the literature [103].

**Figure 8:**
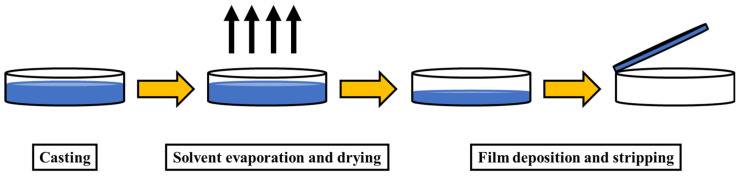
Cast-drying method for nanocomposite film formation.

In comparison to LbL assembly, the one-pot-directed assembly approach overcomes significant problems by being relatively straightforward and quick. This process is used to produce bionanocomposites with high levels of material compatibility, enhanced mechanical properties, and homogeneity [104]. The bionanocomposites prepared through one-pot-directed assembly are thick, similar to microscopic paper, and have high dimensions, but composition control cannot be precise. One-pot-directed synthesis is the most viable technique, as it gives excellent properties to the nanocomposite and takes less time to complete [105]. Cast-drying and vacuum-assisted filtration are two approaches for one-pot-directed synthesis. The use of these two techniques, with the mixture of nanofiller dispersion and biopolymer solution, can successfully prepare multilayer nanocomposite films [106,107]. Vacuum-assisted filtration involves passing a colloidal mixture through nanofilters. Nanofilters allow the solvent to pass through, while trapping the nanofillers that would otherwise cause the polymer to aggregate [108]. For cast-drying, materials are deposited on the surface of the substrate using some specific coating procedure, followed by the evaporation of the solvent, resulting in the deposition of a solid film onto the surface [109]. Figure 8 shows the schematic for the cast-drying process.

Another cost-effective method for cellulose nanocomposite preparation is inkjet printing. In this method, the solution that needs to be coated erupts into tiny drops of liquid and is deposited onto the target with predetermined patterns [110]. Inkjet printing can be used for various liquid “inks”. The resolution and performance of the final product can be adjusted by changing the substrate, droplet size, and viscosity of the solution. To deposit proteins, cell patterns, and DNA with high resolution, various biopolymer materials were employed by the researcher [111,112].

## 6. Applications of Nanocellulose and Its Composites

**Figure 9:**
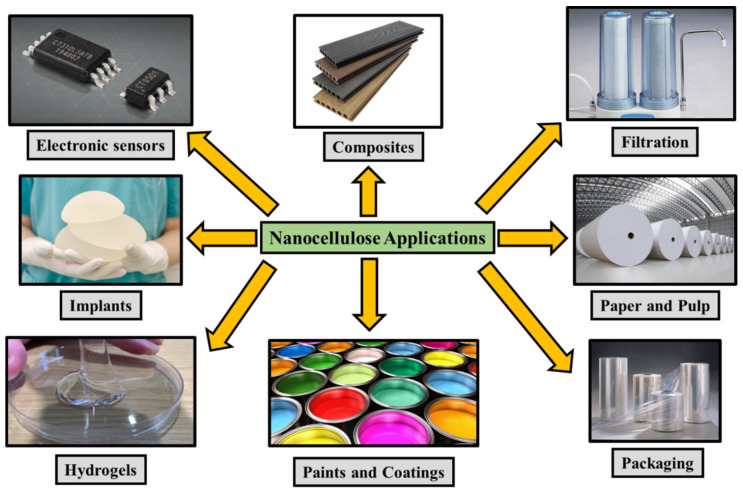
Applications of nanocellulose and its composites.

Nanocellulose is appealing for applications in various industries, such as a thickening agent in cosmetics, a filler in fabrics, and an oil recovery agent. Due to its exceptional characteristics and biodegradability, it is also used in the preparation of transparent paper and nanocomposites with unique functions [19]. Nanocellulose is used to create nanocomposite materials that have excellent thermal conductivity, high mechanical strength, transparency, and are lightweight [15]. Nanocomposites based on cellulose have several applications, as shown in Figure 9. Nanocellulose has been used to create windmill blades with highly durable structures, light armor, flexible batteries, and other various products [113,114]. According to research by Wang et al. [115] on the mechanical performance of nanocomposites reinforced with soybean-extracted nanocellulose, the tensile strength of the polymers is significantly improved, when compared with virgin polymer. Reinforcement of 5 wt. % of cellulose nanofibers in polyvinyl alcohol enhanced the tensile strength from 21 MPa to 103 MPa. The addition of nanofibers changes the stress–strain behavior of the composite, thus improving the overall properties. 

### 6.1. Nanocellulose Based Paper

The paper industry has largely accepted nanocellulose and its related biocomposites for prospective applications. The paper industry primarily uses nanocellulose-based applications to replace petrochemical products [14]. Paper made from nanocellulose has good mechanical properties and is bendable, transparent, and optically clear. Instead of using ordinary paper sheets, these transparent papers can be used for flexible circuits, flexible displays, and electrical devices [116,117]. When Nogi et al. [118] prepared the transparent nanocellulose paper using wood flour, the prepared paper had the same chemical constituents as that of conventional paper, but the difference existed in the fiber sizes and interstitial cavities present. The transparency, high Young’s modulus, and low thermal expansion of the paper make it ideal for electronic devices. Nanocellulose is used in the production of paper as a substrate or as an additive. Among other nanocellulosic materials, cellulose nanofibers are mostly used for paper manufacturing, as they induce special properties to the paper, such as dry strength, low thermal expansion, and low surface roughness. 

Energy supply and its conversion are the essential factors that protect the environment, and they are unquestionably major challenges that humanity has to overcome in this century. Cellulose has high specific modulus, low toxicity, and natural abundance, which makes them the most suitable material for energy application [119,120,121,122]. Due to their increased flexibility and conductivity, nanocellulose paper is abundantly used in the electronics industry. Polyaniline nanocellulose composite films have been extensively researched for a variety of electronic end uses, including paper-based sensors, and flexible electrodes [123,124]. Razaq et al. [125] stated that electrodes used in paper-based energy storage devices can be made by combining polypyrrole, nanocellulose, and carbon filaments. It was observed that the non-electroactive carbon filaments minimized the resistance of the polypyrrole composites. The use of polypyrrole, carbon filament, and nanocellulose offers a substantial advancement in the creation of low-cost, green energy storage units for high-power applications. Additionally, flexible organic electronics can be created using nanocomposite membranes made of highly electrically conductive BNC and undoped poly-(3,4) ethylene dioxythiophene [126]. 

### 6.2. Biomedical Applications

Nanocellulose finds a variety of applications in the medical industry, owing to its biodegradability, low toxicity, and great physical qualities [127,128,129,130]. When utilized as a wound dressing by Hakkarainen et al. [131], they discovered that NFC works as an extremely biocompatible material with the wound donor sites, and the dressing can easily be removed after the skin is healed [132,133,134]. Recent years have seen an increase in research on the uses of nanocellulose in medicine, including medication delivery to specific cells, soft tissue implants, and others [135]. A novel type of hairy cellulose nanocrystalloid, made of CNC with functionalized chains at both ends, is also in high demand for its exceptional properties. By using a chemical process, Hosseinidoust et al. [136] separated the nanocellulose from softwood pulp sheets. Their technique generates nanocrystals with an incredibly high carboxyl content (6.6 mmol g^−1^) and allows for continuous control of the surface charge, without modifying the reaction conditions. Nanocellulose composites are also being used as a scaffold for tissue engineering [137].

The most effective biomedical application of nanocellulose is nanocellulose-reinforced hydrogel composites, having the potential to improve the mechanical performance of polymeric gel formulations with the desired characteristics of reinforcement and matrix [138]. A novel hydrogel with a semi-interpenetrating polymer network having the ability to increase pH sensitivity and excellent mechanical capabilities has recently been developed [139]. This hydrogel can be employed for innovative pharmacological and gene delivery applications. CNC, with the involvement of carboxylated chains, can resist agglomeration and can be taken up by different cells, making it suitable as a carrier for nanomedicine. The use of cellulose in hydrogels increases the shear modulus of the gel, along with a reduction in gelation time and enhanced cell adhesion, thus increasing their use in the biomedical field. Nanocellulose hydrogels can mimic the extracellular matrix at low cytotoxicity in the 3D cell culture. These gels have shown excellent cell regeneration, while providing the necessary mechanical properties for tissue engineering scaffolds, making them useful for treating wounds and repairing cartilage. The use of nanocellulose as an encapsulation for carrying drugs to certain parts of the body is also possible. 

### 6.3. Food Packaging

Effective packaging offers the quality and safe preservation of the food during transportation and storage. The need for biodegradable packaging materials, which cause very little environmental threat and are made from environmentally friendly and renewable resources, has increased. Using three alternative methods of modification, Yang et al. [140] created translucent films from electrosterically stabilized nanocrystalline cellulose (ENCC). The films that were produced by protruding ENCC chains with carboxyl groups displayed 87% light transmission. Furthermore, after being treated with trichloromethyl silane, the transparent films showed prominent hydrophobicity. These organic films can be used to make biodegradable items, such as flexible packaging. Additionally, because they are derived from a natural material, they possess both cost-effective and non-toxic nature, making them an excellent choice for applications involving food packaging [141,142]. Resistance to penetration of oxygen in food packaging is crucial, since, in the presence of oxygen, aerobic microbes start to contaminate the food and destroy its nutritional value. Maintaining a low-oxygen environment is necessary to increase the food’s shelf life. To create a dense network for preventing the penetration of gases, nanocellulose can make hydrogen bonds with itself and with other biopolymers. A dense network is necessary for achieving the gas barrier properties in films used for packaging. Trifol et al. [143] produced nanocomposite film for food packaging applications using nanoclay and nanocellulose as a reinforcement in the PLA matrix. The use of nanocellulose, along with nanoclay, further enhances the water and oxygen barrier ability of the composite film. Packaging with antibacterial properties is also beneficial. The goal of antibacterial active packaging is to maintain and lengthen the shelf life of food products by reducing the growth of bacteria. Therefore, the use of nanocellulose is recommended for making antibacterial packaging because the high surface area of nanocellulose ensures the high loading of the antibacterial material.

### 6.4. Water Treatment

**Figure 10:**
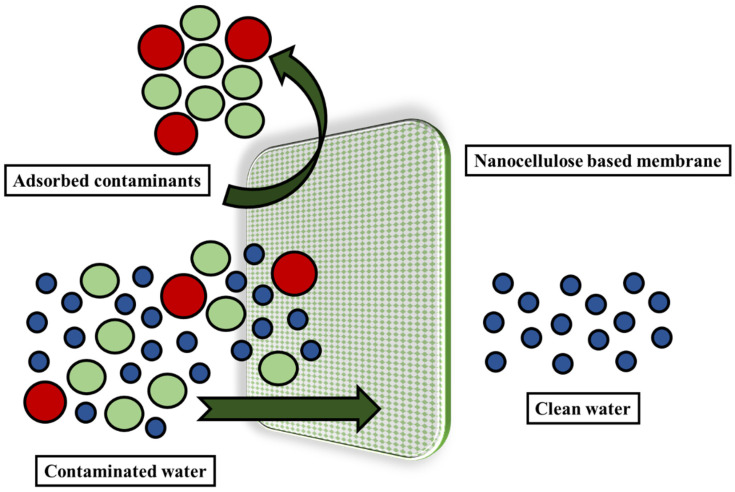
Schematic showing nanocellulosic membranes used to remove water contaminants.

In addition to having biodegradable qualities, nanocellulose are sustainable biomaterials that have a high surface area, numerous reactive sites, and scaffolding stability to support inorganic nanoparticles. These nanomaterials offer a vast range of applications for water treatment. Nanocellulose, when used as reinforcement, has unique characteristics, such as a nanoscale dimension, which gives strength to the composite and provides water and gas barrier properties to it [144]. These properties can be beneficial in creating nanocomposite films with a strong framework for penetrating molecules [145]. Due to the renewable adsorption capacities, composites based on nanocellulose are of tremendous interest in wastewater treatment. Numerous hydroxyl groups on the surface of nanocellulose allow for a wide range of chemical changes. These surface properties of nanocellulose are of great importance and help in designing an environmentally friendly cellulosic membrane to remove impurities from water. Figure 10 shows the schematic for the nanocellulose-based membranes that are used for water treatment. They are the best choice as an adsorbent for the removal of toxins, such as the heavy metals, organic colors, oils, and pharmaceuticals from the moist environment. The adsorption process depends heavily on the interaction between the adsorbent and the adsorbate [146,147]. Different mechanisms, including ion exchange, dipole–dipole contact, complexation on surfaces and pores, van der Waals forces, and hydrophobicity, can cause the interactions between the absorbent and adsorbate. Natural organic materials that are obtained from the degradation of biomass can also have a negative impact on both human health and the environment. Humic acid (HA) and fulvic acid are some of the common acids obtained from biomass that need to be prevented from affecting the water. Humic acid elimination from wastewater was investigated by Jebali et al. [148] using nanocellulose that has been modified by the amine group. They also discovered the involvement of electrostatic forces during humic acid adsorption. The amine group of the amine-modified nanocellulose interacted with the carboxyl and hydroxyl functional groups of the acid to change its behavior [149].

### 6.5. Coatings

Nanocellulose usage in the development of nanocomposite coating and films has been a point of attention for many authors [132,150,151]. Among various applications of nanocellulose, the use of nanocellulose for coating surfaces that have different structures and compositions is also one of the major applications. Variables such as porosity and thickness of the deposited cellulose can be changed to alter the coatings’ permeability and filtering capabilities. For many applications, the mechanical stability of nanocellulose coatings, in both the wet and dry states, is crucial. The first report on the improvement in mechanical characteristics of nanocomposite coatings using cellulose nanofibers as reinforcement and potato starch as the polymer matrix was provided by Dufresne et al. [12]. He described that these nanoparticles have great potential to be used as reinforcement in nanocomposites, due to their impressive mechanical characteristics, reinforcement capacity, low density, and biodegradability. Having a Young’s modulus in between 100–130 GPa and a surface area of a few nanometers, they significantly enhanced the mechanical properties of the polymers, even at low filler loadings. Zimmermann et al. [152] used hydroxypropylated cellulose and observed the enhancement in tensile properties, whereas Nakagaito and Yano [153] examined the same improvement in tensile characteristics by using phenol-formaldehyde resin with nanofiber cellulose (HPC). 

The significance of nanocellulose coatings is highlighted by their high biodegradability, affordability, and availability. To generate self-cleaning surfaces, researchers have been trying to imitate the superhydrophobic nature in nanocellulose-based coatings. Superhydrophobicity can be achieved by creating micro or nanostructures thereby increasing the roughness and lowering the surface energy. A decrease in surface energy affects the wetting properties of the coating, resulting in the achievement of superhydrophobicity [154,155]. Nanocellulose coatings are also used to protect the wood from different environmental agents, such as bacteria or microbes in a humid environment. Previously, unsaturated amino acids, lacquer, and chemical treatments have been used to protect the wood, but the use of these chemicals produces some volatile compounds that may produce environmental problems. Therefore, using a nanocellulose coating that has no environmental impact is recommended [156,157].

## 7. Conclusions and Perspectives

Nanocellulose possesses many exceptional qualities, but its extraction from lignocellulosic biomass is still a significant challenge. To extract the nanocellulose from the biomass, the removal of lignin, hemicelluloses, and other non-cellulosic materials is required, which can be achieved by pretreatment. Pretreatment can involve the use of toxic chemicals; therefore, care should be taken to use eco-friendly chemicals for pretreatment. Nanocellulose extraction techniques also have some limitations, such as obtaining plenty of acidic wastewater during acid hydrolysis, significant energy usage, and prolonged reaction times during enzymatic hydrolysis. Extracted cellulose can then be used as a reinforcement for cellulose-based nanocomposites. Composites based on nanocellulose have excellent qualities and are proven to have great potential in the field of packaging, electronics, and biomedical industries.

This review paper focuses on the introduction of nanocellulose, extraction processes, chemical treatment of cellulose, and nanocellulose-based composites processing, as well as their applications. Composites made from nanocellulose can be used in the near future to make implants, structural components, self-healing materials, protective textiles, etc. Based on the review and arising environmental concerns, it is suggested to use renewable biopolymer sources for industrial and domestic applications that can be beneficial to health care and act as food preservation materials to solve the problems related to food insecurity.

## Figures and Tables

**Table 1:** Types of nanocellulose materials.

Nanocellulose Types	Sources	Extraction Method and Size
CNC	Cotton, tunicin, mulberry bark, hemp, wood, wheat straw.	Acid hydrolysis 5–70 nm in diameter 100–250 nm in length
BNC	Sugars and alcohols.	Extracted from bacterial synthesis 20–100 nm in diameter
NFC	Wood, hemp, flax, potato tuber, sugar beet.	A mechanical method of breaking the cellulose 5–60 nm in diameter

**Table 2:** Nanocellulose isolation methods.

Biological Methods	Mechanical Methods	Chemical Methods
Fungi treatment	Steam explosion	Ionic treatment
Bacteria treatment	Ball milling	Alkaline treatment
Enzymatic hydrolysis	Disintegration	Acid hydrolysis
	Grinding	Oxidation
	Electrospinning	Solvent extraction
	Ultrasonication	
	Homogenization	

**Table 3:** Nanocellulose treatment methods and characteristics.

Modification Method	Chemical Sources	Modified Characteristics	References
Silylation	Alkoxy silane, triethoxyvinylsilane, chlorodimethyl isopropylsilane	Hydrocarbon chains in silane enhance the wettability of cellulose.	[65]
Esterification	Aromatic and aliphatic carboxylic reagents (acidic anhydride)	Plasticization of lignocellulosic strands due to interaction of OH groups of cellulose with acetyl moieties.	[66]
Carbamylation	Isocynaic acids (Butyl 4-(Boc-aminomethyl) phenyl isothiocyanate)	Bonding of functional groups of cellulose with isocyanic acid.	[67]
Functionalized reactions	TEMPO oxidizers (sodium hypochlorites)	Attachment of carboxyl groups on the cellulose surface to initiate further reactions.	[68,69]

## Data Availability

Not applicable.
